# The temporoparietal junction and awareness

**DOI:** 10.1093/nc/niy005

**Published:** 2018-03-27

**Authors:** Michael S A Graziano

**Affiliations:** Department of Psychology and Neuroscience, Princeton University, Princeton, NJ 08544, USA

**Keywords:** awareness, psychophysics, attention

## Abstract

Visual attention and awareness can be experimentally separated. In a recent study (Webb *et al.*, Cortical networks involved in visual awareness independently of visual attention. *Proc Natl Acad USA* 2016a;113:13923–8), we suggested that awareness was associated with activity in a set of cortical networks that overlap the temporoparietal junction. In a comment, Morales *et al.* (Measuring away an attentional confound? *Neurosci Conscious* 2017;3:doi:10.1093/nc/nix018) suggested that we had imperfectly controlled attention thereby jeopardizing the experimental logic. Though we agree that attention behaves differently in the presence and absence of awareness, we argue it is still possible to roughly equate the level of attention between aware and unaware conditions, and that an imbalance in attention probably does not explain our experimental results.

In 2016, my colleagues and I published an experiment that examined brain activity during a visual task (Webb *et al.* 2016a). We attempted to determine which networks in the human cortex were active in association with visual awareness. Participants were exposed to two conditions. In one, the participants reported being subjectively aware of a visual stimulus, whereas in the other, due to a 50 ms tweak to the timing of a mask, the same visual stimulus dropped beneath reportable awareness. We independently measured attention drawn to that stimulus, and found it similar between the two conditions. By manipulating awareness, while holding attention as constant as we could, we hoped to identify brain regions associated with awareness independently of attention. The results implicated specific cortical networks including parts of the fronto-parietal control network and the ventral attention network, both of which overlap the temporoparietal junction (TPJ).

Recently, Morales *et al.* (2017) published a critical commentary on our experiment. Here, I reply to the comment. I will not reply to every detail, but instead focus on the basic concern.

In essence, the concern is that equating attention between the two conditions is tricky. Though we measured attention at one time point (180 ms) after the stimulus onset, we did not measure other time points, and therefore left open the possibility that attention was not fully equated between the two conditions. Perhaps the activity, we obtained was caused by differing levels of attention rather than by differing levels of awareness.

I address three points.

First, it is important to note that the concern is legitimate. My colleagues and I were well aware, and readers should be aware, that equating attention between two conditions is approximate. For this reason, we addressed the issue in the supplementary material of our publication.

In that supplementary material, in a section called “attention confound,” we acknowledged that attention may not have been perfectly equated, and we examined whether a small imbalance in attention could have caused the pattern of results. To address the issue, we performed an additional analysis. We estimated how the amount of attention varied from subject to subject. For some participants, attention appeared to be greater in the aware condition. For others, attention was measured as greater in the unaware condition. We then asked whether this variance was related to the activity obtained in the networks of interest. We found no relationship. An imbalance of attention did not explain the activity in those brain regions.

My second point concerns a specific comment. Morales *et al.* suggested that our reaction-time measure of attention could have been supplemented. They stated that, “attentional effects can also be measured not in terms of reaction times but by target discrimination accuracy.” Yet we reported that discrimination accuracy was not significantly different between the aware and unaware conditions, again suggesting that overall attention was at least roughly comparable between the two trial types.

My third point concerns an earlier behavioral experiment. In that previous study (Webb *et al.* 2016b), we used a behavioral paradigm to manipulate awareness of a visual stimulus while measuring attention to the stimulus. We measured a time course of attention over a period of 590 ms after stimulus onset. The amount of attention varied over that time course. The fluctuations were significantly greater when the participants were unaware of the stimulus than when they were aware of it. We argued that awareness of a stimulus helps to stabilize attention on that stimulus, in accordance with our hypothesis that awareness acts as the internal control model for attention (Webb and Graziano 2015).

In their critique, Morales *et al.* correctly note that in our previous study, attention follows a different time course in the aware and the unaware conditions. They conclude that, therefore, by our own data, attention cannot be considered identical between the aware and unaware conditions, and thus in our brain imaging study, the two conditions cannot be considered to have balanced attention.

I appreciate the concern. Yes, it is impossible to make attention truly identical between the aware and the unaware conditions. However, as shown in Fig. 2A of our behavioral paper (Webb *et al.* 2016b), the overall amount of attention is similar between the aware and unaware conditions. The fluctuations are greater in the unaware case, but the overall magnitude averaged over the time course is roughly similar in the two conditions (it is not significantly different). It was precisely for that reason that we chose a follow-up study, transferring the same behavioral paradigm into the scanner to measure brain activity (Webb *et al.* 2016a). Since attention had been measured to be similar in overall magnitude between the two conditions, but awareness was starkly different, present in one condition and absent in the other, the paradigm provided an opportunity to test whether awareness might drive the activity of specific brain networks.

In a brain imaging study, because many trials are needed per condition, one cannot test very many conditions. Therefore, in our imaging study (Webb *et al.* 2016a), we sampled attention at one time point rather than measuring the entire time course. We had already measured the time course for the paradigm in our previous study (Webb *et al.* 2016b), and therefore a repeat demonstration was unnecessary. This choice of ours in the imaging study, to spot-test attention at only one time point, appears to be the crux of the critique against our experiment. However, since we had already established the time course in a previous study, the criticism seems to us more rhetorical than substantial.

At its heart, the concern raised by Morales *et al.* (2017) seems to be that in our behavioral paper, we argue that attention depends on awareness. Without awareness, the dynamics of attention change. Yet in our brain imaging experiment, we argue that we can find a network that is relatively associated with awareness, independently of attention. Thus Morales *et al.* find what they consider to be a contradiction. Are attention and awareness interdependent, or are they separable? How can they be both? To give an analogy: the motor cortex controls the arm—it helps to stabilize it and direct it. Yet I can still anatomically and functionally distinguish the brain from the arm. Though interacting, they are still separable elements of a larger system. To argue that they must be either interacting, or separable, but not both, is not valid. Just so, my colleagues and I proposed the provisional theory that the brain constructs awareness as an attention schema—a representation that monitors and to some extent predicts attention (Webb and Graziano 2015). That attention schema plays a crucial role in controlling attention. The two processes interact. Change one, and you change the other. And yet it should still be possible to look for separable anatomical bases for them. Keep one (attention) roughly the same, while varying the other (awareness) to one extreme or the other, and we find that a specific brain network is indicated (Webb *et al.* 2016a).

In conclusion, of course I agree that our brain imaging study is only one crack at the question, from one methodological angle, and no scientist should believe the story without convergent evidence. So far, at least some convergent evidence points to visual awareness associated with cortical networks that pass through the TPJ. In hemispatial neglect, damage to the TPJ classically causes a severe disruption of awareness (Vallar and Perani 1986). In brain imaging studies of visual awareness, the TPJ, especially its dorsal part, often shows robust activity even if it is not emphasized in the text of every paper (e.g. Lumer *et al.* 1998; Dehaene *et al.* 2001; Naghavi and Nyberg 2005; Carmel *et al.* 2006; Persaud *et al.* 2011; Bor and Seth 2012; Cortese *et al.* 2016). However, I caution against over-interpreting specific brain areas. A network approach is more realistic (Igelström and Graziano 2017). I stand by the argument that, thus far, increasing evidence points to the TPJ as an important network node related to awareness, both one’s own awareness and attributing the property of awareness to others, without undercutting arguments for other nodes.

## Funding

Princeton Neuroscience Innovation Fund.

*Conflict of interest statement*. None declared.
